# Climate Change and Its Health Impact in South Africa: A Scoping Review Protocol

**DOI:** 10.3390/ijerph22071155

**Published:** 2025-07-21

**Authors:** Olubunmi Margaret Ogbodu, Ayodeji Oluwabunmi Oriola, Busisiwe Mrara

**Affiliations:** 1 Department of Anaesthesiology and Critical Care, Faculty of Medicine and Health Sciences, Walter Sisulu University, Mthatha 5117, South Africa; oogbodu@wsu.ac.za (O.M.O.); bmrara@wsu.ac.za (B.M.); 2 Department of Chemical and Physical Sciences, Faculty of Natural Sciences, Walter Sisulu University, Mthatha 5117, South Africa

**Keywords:** climate change-induced health impacts, vulnerable populations, climate action policies, environmental health, health disparities, South Africa

## Abstract

Climate change is profoundly impacting human health in South Africa, aggravating existing health challenges and creating new threats, particularly in vulnerable populations. This scoping review aims to comprehensively map existing evidence of climate change and diverse human health impacts to assist in the equipping of health systems to address evolving challenges of climate change. The scoping review will inform the development of evidence-based policy, improve public health preparedness, and ensure that adaptation strategies are effectively tailored to South Africa’s socio-economic and environmental conditions. This scoping review protocol will be conducted using the Joanna Briggs Institute (JBI) methodology, following five steps: (1) defining the research question, (2) search strategy, (3) setting inclusion criteria, (4) extracting data, (5) assessing, summarizing, and presenting findings. The Preferred Reporting Items for Systematic Reviews and Meta-Analyses for Scoping Reviews (PRISMA-ScR) tool will be used. A comprehensive peer-reviewed literature search, including PubMed, Scopus, ScienceDirect, and Google Scholar, will be conducted by two independent reviewers. The review will be conducted over eight weeks, focusing on English studies published between 2015 and 2025, and conducted within South Africa. A two-stage screening process will determine article eligibility. Disagreements will be resolved through consensus and consultation of a third reviewer. The results of this review will be presented as tables, including a narrative synthesis of the findings.

## 1. Introduction

Climate change is an urgent global challenge, with rising temperatures and extreme weather events impacting human health significantly [1]. Extreme weather conditions can directly lead to physical injuries and deaths or indirectly heighten the risk of heat-related illnesses [1,2,3]. These health-related illnesses include debilitating conditions arising from heat exhaustion and heatstroke, particularly among vulnerable populations [1,2,3]. Similarly, increasing temperatures significantly heightens the risk of heat-related deaths, particularly among vulnerable groups like the elderly, children, and individuals with pre-existing health conditions, who are less capable of maintaining proper body temperature regulation [4,5]. It could also lead to an increase in excruciating conditions such as heart-related and respiratory diseases, as well as mental health challenges [6,7,8]. These increase mortality rates, leading to more significant public health concerns [6,7,8]. Heat-related illnesses can often be prevented through effective planning and implementation of targeted adaptation strategies. These strategies include improved urban design, access to cooling centers, and public health education. However, the success of these measures depends on adequate resources, infrastructure, and community engagement, which may be lacking in vulnerable regions. Additionally, these strategies may not fully address climate change’s broader systemic challenges, thus highlighting the need for global action to mitigate its impacts [9,10]. Although climate change’s health impacts vary significantly across populations and regions, its occurrence will resonate similarly [11]. This is as individuals and groups will continue to experience different levels of exposure, sensitivity, and adaptive capacity, collectively shaping their vulnerability. These underscore the urgent need to address climate change as a critical health concern that disproportionately affects the most vulnerable. It is also worth noting that climate change acts as a threat multiplier, thus intensifying human health risks. This, therefore, amplifies existing vulnerabilities and undermines health systems’ capacity to provide consistent quality care [12,13]. Given the diverse impacts of climate change on human health, it is imperative to prioritize research that establishes clear linkages between environmental changes and health outcomes. Eventually, this escalating strain on healthcare facilities will disproportionately affect regions with weak health infrastructure and limited adaptive capacity, where the impacts of climate change are most challenging to manage [12,14]. The World Health Organization (WHO) identifies climate change as a significant global health threat and, as a matter of urgency, intensifies efforts to address its impacts. The health system faces mounting pressure as it struggles to respond to the increasing burden of climate-related illnesses while simultaneously addressing other infectious diseases, such as tuberculosis and HIV/AIDS.

South Africa’s unique geography and climate make it particularly vulnerable to the impacts of climate change [15,16]. Its diverse landscape, spanning coastal regions, mountainous areas, and arid deserts, creates various distinct climate zones, each with its challenges [15,16]. The country’s diverse geography across nine provinces creates varied climates, from the Mediterranean climate of the Western Cape to KwaZulu-Natal’s humid subtropical conditions [15,16]. While most provinces experience summer rainfall, the Western Cape receives winter rainfall. Thunderstorms are common in Gauteng, North West, and KwaZulu-Natal, while the Eastern Cape features hot, dry summers and cold, moist winters [15,16]. South Africa’s climate ranges from Mediterranean in the southwest to temperate on the interior plateau and subtropical in the northeast, offering mild conditions year-round and abundant sunshine [17,18,19]. This variability exposes the country to a broad spectrum of climate-related hazards, including rising sea levels, droughts, heat waves, and intense storms, amplifying the need for targeted adaptation and resilient strategies. These climate-related hazards become worrisome as rising temperatures and changing rainfall patterns exacerbate the country’s health issues [20,21]. South Africa’s diverse climate conditions, intensified by climate change, significantly impact human health through various pathways. For example, rising temperatures and altered rainfall patterns contribute to the spread of disease vectors, such as mosquitoes, increasing the risk of vector-borne diseases [22,23,24]. Climate changes also negatively impact water quality, affecting food security and access to clean water, giving way to water-borne illnesses [22,23,24]. These interconnections between South Africa’s climate conditions and human health also highlight the critical link between environmental health and human well-being. Significant barriers to adaptation constrain South Africa’s efforts to mitigate the health impacts of climate change [24]. This is despite policies promoting renewable energy, policy uncertainty, corruption, and inadequately prepared health systems, which often slow the country’s response [24,25]. Key challenges include insufficient financial resources, limited human capacity at provincial and local levels, and weak political will at the local level [26,27,28]. Poor coordination across government sectors, the absence of climate change units at district and local levels, and a lack of up-to-date information in Integrated Development Plans (IDPs) further impede progress [26,27,28]. Additionally, communities often lack an understanding of climate adaptation, and local staff responsible for environmental duties frequently have insufficient expertise [26,27,28]. These systemic challenges exacerbate vulnerabilities, making it difficult to address the growing health risks associated with climate change.

### 1.1. Rationale for Scoping Review

In South Africa, a scoping review protocol on climate change and health is particularly valuable due to the country’s diverse geography, unique climate, and vulnerability to climate-related hazards. However, in the face of this complexity and the interdisciplinary nature of climate change and health interactions, a scoping review protocol will help define clear objectives, inclusion criteria, and methodological approaches, facilitating research collaboration, providing a roadmap for this study, and ensuring that the findings contribute meaningfully to policy development and future research on climate-related health impacts. Despite increasing research in this area, a comprehensive synthesis of existing studies is needed to identify knowledge gaps, guide evidence-based decision-making, and inform policy. Policy responses may be inadequate or misaligned with the country’s healthcare priorities without comprehensive research. By incorporating diverse evidence, including gray literature, a scoping review can offer a holistic understanding of the intricate linkages between climate change and human health, enabling targeted and effective interventions. This review will also enhance knowledge translation by making research findings accessible to key stakeholders, including policymakers, practitioners, and researchers. Additionally, this scoping review protocol will lay the groundwork for scoping and systematic reviews that can delve deeper into specific topics, such as the effects of climate change on vector-borne diseases or the efficacy of climate adaptation strategies in South Africa.

### 1.2. Methods and Analysis

The main research question for this review is the following: What is known from the literature about the impacts of climate change on human health in South Africa?

The research objectives are as follows:

1. Assess the current state of knowledge on the impacts of climate change on human health in South Africa.
2. Describe South Africa’s most vulnerable populations to the health impacts of climate change.
3. Examine the existing climate change adaptation strategies and policies in South Africa and examine the effectiveness of the health policies and adaptation strategies in mitigating health risks in South Africa. The effectiveness will include strengthening disease prevention and health promotion efforts, including those related to climate-related diseases.

## 2. Materials and Methods

### 2.1. Methodology

The reviewers (O.M.O., A.O.O., and B.M.) will use the Joanna Briggs Institute (JBI) methodology to conduct this study, guided by the Preferred Reporting Items for Systematic Reviews and Meta-Analyses extension for Scoping Reviews (PRISMA-ScR) tool and checklist [29,30,31]. The choice of JBI methodology by the authors was made because it provides a structured framework that ensures transparency, reproducibility, and rigor in scoping reviews. It provides a comprehensive and systematic approach to evidence synthesis while the PRISMA-ScR tool is a checklist that helps authors, reviewers, and readers understand the key elements that should be included in a scoping review’s methodology and results. This approach ensures the inclusion of the literature from diverse study designs, encompassing both peer-reviewed research and gray literature. This tool supports the comprehensive reporting of methods and findings and helps improve the quality of scoping reviews by enhancing transparency and uniformity in reporting.

This study will be conducted in five steps: (1) defining the research question, (2) developing a search strategy, (3) establishing inclusion criteria, (4) extracting data, and (5) data assessment, summarizing and presenting the results. Quality assessment is regarded as a critical element of scoping studies, enabling reliable research evidence to be disseminated to a larger audience in a way that is useful to practice and policymaking and for future researchers [32]. This scoping review protocol was not registered; nonetheless, it will be published to ensure access to a large audience.

### 2.2. Formulating the Research Question

Given that a well-formulated research question ensures that a scoping review systematically explores the relevant literature, identifies knowledge gaps, and synthesizes evidence meaningfully, clearly defined objectives and the scope of this review were identified. These enable the framing of the research questions. These guide the search strategy, inclusion criteria, and data analysis, ensuring that the scoping review addresses finding the link between climate change and health impact in South Africa. Hence, the following research questions will be answered in this scoping review:

▪ In what ways is climate change impacting on human health in South Africa?
▪ Who are the most at-risk populations for climate change and health in South Africa?
▪ What climate change adaptation strategies and policies in South Africa are effective for addressing the health impacts of climate change?

### 2.3. Developing a Search Strategy

The search will be conducted using a research-librarian-led electronic search strategy and following reviewers’ careful consideration. A comprehensive search strategy will be developed using PubMed, Scopus, ScienceDirect, and Google Scholar electronic databases for peer-reviewed articles and a gray literature search focusing on English-language studies published between 2015 and 2025. For the electronic database search, a carefully developed combination of keywords and search terms will be employed to ensure the inclusion of the most relevant studies, following thorough consideration by the review team. The specific keywords or Medical Subject Headings (MeSH) terms and Boolean operators (“OR/AND”) will be applied. These search terms will include the study keywords and phrases such as “climate change AND health in South Africa” OR “health effects OR health impacts of climate change in South Africa” AND “vulnerable populations to climate change and human health impact in South Africa”. Furthermore, relevant journals and publications will be searched manually to identify any studies that may have been missing during the electronic search.

On the other hand, the gray literature search will include government reports, white papers, conference proceedings from relevant events such as the African Climate Change Conference and the South African Health Research Conference and the web pages of the South African Department of Health, the South African Department of Environmental Affairs and the World Health Organization (WHO) will be searched. The selected articles will be compiled and managed using EndNote V.20 (Clarivate) to organize references and eliminate duplicate entries. The study selection process is expected to be finalized within eight weeks, from 1 September to 30 October 2025, following the PRISMA-ScR checklist guidelines [31]. The comprehensive PubMed search string is attached as a Supplementary File. After extracting all relevant records from the databases and removing duplicates, the Covidence platform (Covidence systematic review software (http://www.covidence.org/), Veritas Health Innovation, Melbourne, Australia) will streamline data, facilitating collaboration among the reviewers during the screening stages [33]. The search strategy created in PubMed is shown in Table 1 below.

**Figure 1:**
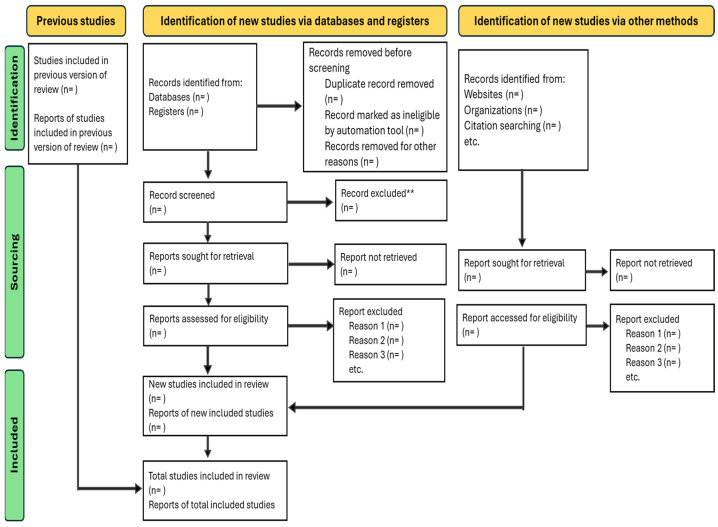
PRISMA-ScR visually represented flow diagram or flowchart illustrating the article selection process [31] and PRISMA extension for scoping reviews (PRISMA-ScR): checklist and explanation.

Excluded studies will be documented, and the PRISMA-ScR flow diagram will present the selection process (Figure 1). The PRISMA-ScR flowchart is shown in Figure 1 in the manuscript. As this is a protocol, the actual database search results are not available and cannot be presented in a flowchart. However, a blank PRISMA-ScR flowchart is shared in the manuscript.

### 2.4. Establishing Inclusion Criteria

Clearly defined inclusion criteria ensure a systematic, transparent, and reproducible selection process in a scoping review. They establish boundaries, capture diverse evidence, and enhance replicability, allowing future researchers to validate findings or expand the review for further exploration [34]. Studies based on the research question and scope are eligible for inclusion if they meet the following criteria, except otherwise excluded. The inclusion criteria will follow the Population, Concept, and Context (PCC) framework detailed below.

#### 2.4.1. Population

The population of interest will be all the diverse ethnic groups in South Africa to examine the link between climate change and human health across South Africa’s diverse geographical distribution, unique climate, and vulnerability to climate-related hazards.

#### 2.4.2. Concept

All climate change-related phenomena and factors that influence health outcomes and their direct and indirect health impacts in South Africa, with emphasis on groups and demographics most at risk from climate change’s impacts, the nexus between climate change and health, and the policy responses, as well as climate-sensitive diseases in South Africa, will be considered.

#### 2.4.3. Context

Any existing or new health risks that climate change would aggravate and associated linkages to South Africans will be considered. All South African provinces with diverse climate change occurrences will be considered.

### 2.5. Inclusion Criteria

Full-text peer-reviewed journal articles including different study designs (qualitative, quantitative, observational, and mixed methods) and gray literature published in English from 2015 to 2025.

### 2.6. Exclusion Criteria

Non-English articles, studies outside South Africa, systematic and narrative reviews outside South Africa, and studies published before 2018.

### 2.7. Study Timeframe

The search is limited to 2015–2025, which aligns with the Sustainable Development Goals (SDGs) era, emphasizing climate action and health as critical global priorities. This study timeframe also considered pre- and post-COVID-19 periods, given the significant interruptions of COVID-19 on climate change and human health. In addition, in 2015, a significant policy focus on climate change and health globally occurred with the adoption of the Paris Agreement in 2015. This agreement, adopted at the UN Climate Change Conference (COP21) in Paris, explicitly acknowledges the link between climate change and health and aims to strengthen the global response to the threat of climate change, including its health impacts. Therefore, the year 2015 is a considered a significant year in relation to climate change and health impacts.

### 2.8. Data Extraction

A standardized data charting form based on the research objectives will be designed to ensure accuracy and comprehensiveness. This form will be pilot-tested on a small sample of studies to ensure its effectiveness. This form will capture study characteristics, climate change exposure, health outcomes, adaptation strategies, and climate action policies. See Table 2 below. Study characteristics will include author, year of publication, study design, location, and population studied in all the South African provinces. Climate change exposure will be recorded based on type, duration, and intensity. Health outcomes will be captured according to type, measure, and prevalence. Adaptation strategies and policies will be recorded in terms of type and effectiveness. Finally, study quality will be assessed using the Joanna Briggs Institute Critical Appraisal Checklist to ensure that only high-quality evidence is considered in this review, thus strengthening the reliability of the findings and conclusions. In this scoping review, the methodological quality of the included studies will be assessed to identify potential biases and limitations, ensuring a comprehensive and credible evaluation of the evidence. Two reviewers (OMO and AOO) will independently extract data from each study using a standardized form to ensure a systematic and transparent process. Any disagreement will be resolved through discussion and consensus, with a third reviewer (BM). The extraction process will occur in two stages: an initial phase, in which both reviewers collect data independently, and a verification phase, in which both reviewers assess completeness and correctness. This structured approach enhances the rigor and reliability of data extraction, ensuring consistency in capturing key information related to the research questions. The inclusion and exclusion criteria for the initial data extraction of this scoping review were assessed using a set of citations randomly selected to ensure consistency. Upon finalization, the inter-rater reliability of another random sample of identified studies will be evaluated using Cohen’s Kappa coefficient [35]. Cohen’s Kappa coefficient provides a data-driven means to validate these reviews, helping researchers and practitioners pinpoint inconsistencies and biases before the results are skewed. This leads to improved trust in the results and enhanced credibility of the review outcomes. Sampling will continue until a kappa score of 0.85 is achieved. To maintain a high level of agreement, inter-rater reliability will be routinely assessed throughout the title and abstract screening process. Beyond Cohen’s Kappa coefficient, discrepancies will be resolved through discussion and consensus between the 3 reviewers (OMO, AOO, and BM); otherwise, an expert in the field will be consulted.

### 2.9. Data Charting Form

Acquired data will be reported as indicated in Table 2.

## 3. Data Assessment, Summarizing, and Presenting the Results

The qualitative and quantitative methods will be used for this assessment. Qualitative analysis will involve a thematic evaluation of the data extraction table to identify key themes within the literature. Thematic analysis will be conducted using the ATLAS.ti 21 software for qualitative analysis.

Data will be organized in tables and graphs to address research questions, guide future research directions, and highlight the most at-risk populations affected by climate change-related health risks in South Africa.

Once data extraction is complete, the findings will be collated, summarized, and reported to provide a comprehensive overview of the current knowledge on climate change’s impact on human health in South Africa to find the nexus. The extracted data will be systematically organized into a table for easy comparison and analysis, categorizing information by study characteristics, climate change exposure, health outcomes, adaptation strategies, and study quality. For quantitative data, descriptive statistics will be used to summarize study characteristics, climate change exposure, and health outcomes. At the same time, a narrative synthesis will provide insights into adaptation strategies and climate action policies. The summarized findings will be presented clearly and concisely using tables, figures, and text. The report will include sections on an executive summary, introduction, methods, results, discussion, and conclusion. The results section will describe the climate change-associated impacts on health in South Africa, highlighting key health outcomes and adaptation strategies in alignment with this review’s objective. The discussion will interpret the findings, identify knowledge gaps, and make recommendations for future research endeavors. The findings will be narratively summarized.

### 3.1. Patient and Public Involvement

Neither patients nor the public were involved in the design, execution, reporting, or dissemination strategy of this scoping review protocol.

### 3.2. Ethics and Limitations

Ethical approval was not obtained for this study as neither patients nor the public were involved in this scoping review protocol. The potential limitations of scoping reviews are a lack of critical appraisal of evidence synthesis, and they often show gaps in the existing research and suggest directions for future studies, rather than providing answers to specific research questions.

### 3.3. Dissemination

The findings will be disseminated to South African policymakers through organized workshops and seminars with management at the municipalities and provincial offices. Research feedback meetings will be organized in the communities most affected by collaborating with the traditional rulers and community-based organizations, as required for effective dissemination and education to achieve positive behavioral changes towards climate action from the community members.

Furthermore, the findings will be shared through peer-reviewed academic publications and at local, national, and international conferences.

### 3.4. Supporting Information

Search Strategy: PubMed and PRISMA-ScR Checklist (File S1).

## 4. Conclusions

This scoping review will map existing evidence on the current state of knowledge on the climate change impacts on human health in South Africa, South Africa’s most vulnerable populations to the health impacts of climate change, the existing climate change adaptation strategies and policies in South Africa, and the effectiveness of the policies and strategies in addressing health impacts. The authors hope that the scoping review, when conducted, may provide some guidance useful as a framework to inform proposals on climate change adaptation strategies relevant to mitigating the negative impact of climate change on human health in South Africa.

## Figures and Tables

**Table 1:** Search Strategy developed for the PubMed database—scoping review protocol on climate change.

Electronic Database	Search Term	Keywords
**PubMed**	(“Climate Change” [Mesh] OR “South Africa *” [tw] OR “epidemiology *” [tw] OR “Environmental Health *” [tw] OR “populations at risk *” [tw] OR “vulnerable populations *” [tw] OR “human health impact *” [tw] OR “health policy *” [tw] OR “climate action*” [tw] OR “wellbeing*” [tw] OR “ climate change-related health impacts*” [tw] OR “Climate adaptation strategies*” [tw] AND “South Africa*” [tw] AND (1 January 2015:29 April 2025 [pdat])) AND (Climate change)	climate change, health impacts, at-risk populations, environmental health, climate action policies, South Africa

* This sign indicates that the search was focused on studies in South Africa.

**Table 2:** Data charting form.

1	Author
2	Publication Year
3	Study Title
4	Study Design
5	Study Outcome
6	South African Province where this study was conducted
7	Vulnerable populations to climate change in the province
8	Climate change adaptation strategies
9	Climate change policy in place
10	Study recommendations

## Data Availability

The current study did not produce or analyze any datasets. Upon research completion, all pertinent data will be made public. There will be a scoping review, and the outcomes will be presented.

## Supplementary Materials

The following supporting information can be downloaded at: https://www.mdpi.com/article/10.3390/ijerph22071155/s1, File S1: Search Strategy: PubMed and PRISMA-ScR Checklist.

## Abbreviations

The following abbreviations are used in this manuscript:

HIV/AIDS	Human Immunodeficiency Virus/Acquired Immune Deficiency Syndrome
IDPs	Integrated Development Plans
MeSH	Medical Subject Heading
PRISMA-ScR	Preferred Reporting Items for Systematic Reviews and Meta-Analyses for Scoping Reviews
SDGs	Sustainable Development Goals
WHO	World Health Organization
