# Supplementary material for: Climate Change and Its Health Impact in South Africa: A Scoping Review Protocol
Source: Int J Environ Res Public Health. 2025 Jul 21;22(7):1155. doi: 10.3390/ijerph22071155 (PMC12294215; doi:10.3390/ijerph22071155)
Supplement: Supplementary file 1 [file ijerph-22-01155-s001.zip › ijerph-3640836-supplementary.pdf]

Supplementary file for Climate change paper: electronic database search strings

Databases searched:

- PubMed/MEDLINE (National Library of Medicine)
- Scopus (Elsevier)
- Science Direct (Elsevier)
- Google Scholar

Electronic database	Search terms
PubMed	("Climate Change"[Mesh] OR "Extreme Weather Events"[Mesh]) AND ("Heatwave"[Title/Abstract] OR "Drought"[Title/Abstract]) AND ("Respiratory Tract Diseases"[Mesh] OR "Asthma"[Mesh])<br><br>("Climate Change" [Mesh] OR "South Africa*" [tw] OR "epidemiology*" [tw] OR "Environmental Health*" [tw] OR "populations at risk*" [tw] OR "vulnerable populations*" [tw] OR "human health impact*"[tw] OR "health policy*" [tw] OR "climate action*" [tw] OR "wellbeing*"[tw] OR " climate change-related health impacts*"[tw] OR "Climate adaptation strategies*"[tw] AND "South Afri-ca*"[tw] AND (2015/1/1:2025/4/29[pdat])) AND (Climate change)
Scopus	("climate change" OR "global warming") AND ("human health" OR "public health") AND ("air pollution" OR "respiratory illness") AND (South Africa) AND (2015-2025)
Science Direct	"climate change" AND "human health" AND ("heat stress" OR "heat waves")"climate change" AND "respiratory illness" AND ("air pollution" OR "wildfires")"climate change mitigation" AND "south africa " AND "adaptation strategies"
Google Scholar	“health impacts of climate change on specific vulnerable populations,<br>“health impacts of climate change on children, the elderly, or those living in poverty”<br>“correlation between extreme heat and increased mortality rates, particularly among vulnerable populations”<br>“health consequences of events like floods, droughts, and wildfires”<br>“Indirect Health Impacts of climate change on humans”<br>“Climate change and access to healthcare”
